# Ivacaftor Inhibits Glioblastoma Stem Cell Maintenance and Tumor Progression

**DOI:** 10.3389/fcell.2021.678209

**Published:** 2021-05-11

**Authors:** Kun Liu, Jun Pu, Zhi Nie, Yulin Shi, Liping Jiang, Qisheng Wu, Yongbin Chen, Cuiping Yang

**Affiliations:** ^1^Key Laboratory of Animal Models and Human Disease Mechanisms of Chinese Academy of Sciences and Yunnan Province, Kunming Institute of Zoology, Kunming, China; ^2^Kunming College of Life Science, University of Chinese Academy of Sciences, Beijing, China; ^3^Department of Neurosurgery, The Second Affiliated Hospital, Kunming Medical University, Kunming, China; ^4^Center for Excellence in Animal Evolution and Genetics, Chinese Academy of Sciences, Kunming, China

**Keywords:** ivacaftor, glioblastoma, glioblastoma stem cell, apoptosis, stemness

## Abstract

Glioblastoma (GBM) is the most common and malignant primary brain tumor. Glioblastoma stem cells (GSCs) not only initiate and sustain uncontrolled cell proliferation but also resistant to conventional clinical therapies including temozolomide (TMZ) dependent chemotherapy and radiotherapy, implying that there is an urgent need to identify new therapeutic strategies especially specific targeting GSCs. Here, we provide evidence showing that ivacaftor commonly applied in cystic fibrosis therapy acts as a potent inhibitor for GSCs maintenance. We found that ivacaftor promotes cellular apoptosis *in vitro* and represses patient-derived xenograft (PDX) tumor growth *in vivo*. In addition, we demonstrate that ivacaftor decreases stemness marker gene expressions of GSCs, including CD133, CD44, and Sox2. In summary, our findings reveal that ivacaftor inhibits glioblastoma progression via specifically eliminating GSCs, which opens a new avenue for GBM clinical therapy in the future.

## Introduction

Glioblastoma (GBM) is the most aggressive primary tumor in the central nervous system (Stupp et al., 2005; Wen and Kesari, 2008; Strickland and Stoll, 2017). Despite conventional clinical therapies including surgery, radiotherapy, chemotherapy, and immunotherapy, the median survival of GBM patients remains less than 15 months after diagnosis (Wen and Kesari, 2008). It has been well recognized that GBM was initiated by a subpopulation of cells termed as glioblastoma stem cells (GSCs), which are characterized by sustaining abilities of self-renewal, multilineage differentiation and resistance to current therapeutic strategies (Chen et al., 2012; Parada et al., 2017). Radiotherapy and chemotherapy are cytotoxic to highly proliferative tumor cells, but fail to eliminate the relatively quiescent GSCs, which is the major cause leads to GBM recurrence (Das et al., 2008; Lathia et al., 2015). Temozolomide (TMZ) is an orally alkylating agent that commonly used for the standard glioma clinical treatment via triggering DNA damage (Stevens et al., 1987; Ochs and Kaina, 2000). However, more than 50% of glioma patients received TMZ treatment show resistance to TMZ therapy (Lee, 2016). This is attributed to GSCs recalcitrance to TMZ therapy due to their enhanced DNA repair ability or/and high methyl guanine methyl transferase expression (Hu et al., 2019; Gao et al., 2021). Small molecule compounds, including clofoctol (Hu et al., 2019), LLP-3 (Guvenc et al., 2013), gboxin (Shi Y. F. et al., 2019), inhibitor of dopamine receptor D4 (Dolma et al., 2016), and inhibitor of STAT3 (Shi et al., 2018), have been documented to inhibit GSCs self-renewal ability. However, so far, none of an optimal drug was used for selectively targeting GSCs approved for GBM clinical therapy. Therefore, there is an urgent need to identify new drugs synergizing with TMZ to effectively eliminate GSCs and translate them into clinical application for GBM patients.

Ivacaftor was approved by U.S. Food and Drug Administration in 2019 for use in cystic fibrosis (Hoy, 2019). The clinical safety of ivacaftor was confirmed by a phase III trial study (Rowe et al., 2017). In this study, we identified ivacaftor as a promising anti-glioblastoma drug via specifically and effectively repressing GSCs. Mechanistically, we provide evidence showing that ivacaftor inhibits GSCs stemness marker genes expressions including CD133, CD44, and Sox2. In summary, our findings reveal that ivacaftor inhibits glioblastoma progression via specifically eliminating GSCs, which opens a new avenue for GBM clinical therapy in the future.

## Results

### Ivacaftor Inhibits GSCs Proliferation and Induces Cellular Apoptosis

**FIGURE 1:**
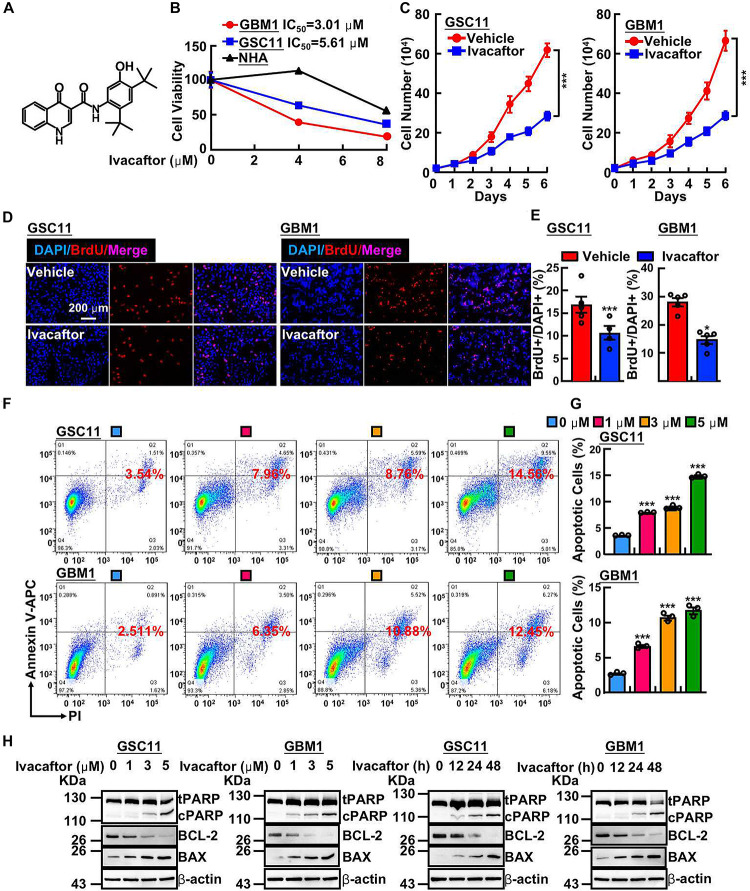
Ivacaftor inhibits GSCs proliferation and induces cellular apoptosis. **(A)** The chemical structure of FDA-approved drug ivacaftor. **(B)** Cell viability of indicated cells treated with various concentrations of ivacaftor. **(C)** The cells proliferation of GSC11 (left panel) and GBM1 (right panel) treated by 5 mM ivacaftor. **(D,E)** Representative images of BrdU incorporation assay in GSC11 (left panel) and GBM1 (right panel) treated with or without ivacaftor. Scale bar: 200 mm. **(E)** Quantification data for **(D)**. **(F,G)** Ivacaftor treatment significantly increases cellular apoptosis in GSC11 and GBM1 cells determined by flow cytometry assay. **(G)** Quantification data for **(F)**. **(H)** Apoptotic cell markers were dramatically increased following treatment with indicated concentrations of ivacaftor. tPARP, total PARP; cPARP, cleaved PARP. Data are shown as means ± SEM, *P < 0.05; ***P < 0.001; *t*-test.

Ivacaftor was generally used for cystic fibrosis therapy, however, its potential activities in anti-cancer have not been characterized. As shown in Figure 1A, the chemical structure of ivacaftor was depicted. To explore the drug repurposing effect of ivacaftor, we examined the potential inhibitory effect of ivacaftor in GSCs, normal human astrocyte cells (NHA) and two GSCs including GSC11 (Bhat et al., 2013) and GBM1 (Zhou et al., 2018) cells, were treated with indicated concentrations of ivacaftor. As shown in Figure 1B, ivacaftor significantly inhibits GSCs cell viability, compared with less inhibitory effect in NHA cells, implying that ivacaftor specifically blocks GSCs survival. The cell proliferation of GSCs was also attenuated following ivacaftor treatment (Figure 1C). To verify whether the cell cycle of GSCs was disrupted by ivacaftor, we performed BrdU incorporation assay. As shown in Figures 1D,E, the cell proliferation was substantially impaired after ivacaftor treatment compared to control group, as indicated by decreased BrdU positive-cell ratio after ivacaftor treatment. Furthermore, the cellular apoptosis of GSC11 cells treated with ivacaftor was determined by flow cytometry experiment, which was gradually induced with increased amount of ivacaftor treatment (Figures 1F,G). In addition, immunoblot results confirmed that apoptotic related factors, including Bax and cleaved PARP, were remarkably increased, while the anti-apoptosis marker, Bcl-2 was decreased, following ivacaftor treatment in a dosage or time-dependent manner (Figure 1H). These findings strongly indicate that ivacaftor inhibits GBM via inducing GSCs cellular apoptosis and attenuating GSCs self-renewal ability.

### Ivacaftor Attenuates GSCs Self-Renewal

**FIGURE 2:**
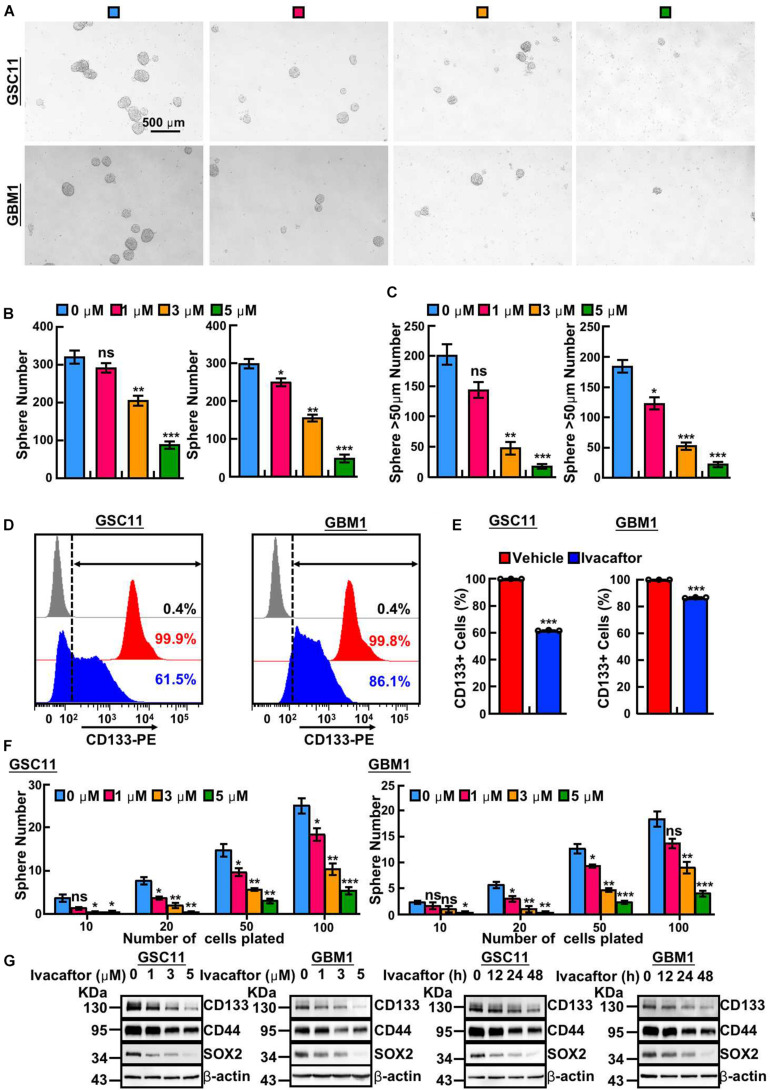
Ivacaftor inhibits GSCs self-renewal ability. **(A)** Representative images of tumor sphere derived from GSC11 (top panel) and GBM1 (down panel) treated with indicated concentrations of ivacaftor. Scale bar: 500 μm. **(B)** Tumor sphere formation ability was significantly attenuated by treating GSC11 (left panel) and GBM1 (right panel) cells with different concentrations of ivacaftor. **(C)** Ivacaftor treatment significantly decreased GSC11 (left panel) and GBM1 (right panel) larger tumor sphere (diameter larger than 50 μm) formation ability. **(D,E)** CD133 membrane accumulated positive cells in GSC11 and GBM1 were decreased after treatment with 5 μM ivacaftor for 48 h determined by flow cytometry assay. **(E)** Quantification data for **(D)**. **(F)** Effects of ivacaftor in indicated cells evaluated by *in vitro* limiting dilution assay. **(G)** GSC11 and GBM1 cells self-renewal ability and stemness marker gene expressions were inhibited by ivacaftor via time- or dosage-dependent manner. Data are shown as means ± SEM, *P < 0.05; **P < 0.01; ***P < 0.001; *t*-test.

To further investigate the inhibitory effects of ivacaftor on GSCs, we took advantages of tumor sphere formation approach to examine the GSCs self-renewal ability. We found that ivacaftor caused a dose-dependent suppression of tumor spheres formation ability of GSCs. As shown in Figures 2A–C, ivacaftor treatment decreased tumor sphere formation especially larger tumor spheres (diameter larger than 50 μm). Consistently, GSC11 cells were treated with or without 5 μM ivacaftor followed by staining with CD133 antibody to validate the membrane accumulated CD133 expression, our results showed that CD133 positive cells were markedly decreased by ivacaftor treatment compared with control (Figures 2D,E). Consistently, the *in vitro* tumor sphere limiting dilution assay showed that the tumor spheres were dramatically decreased after ivacaftor treatment (Figure 2F). Furthermore, the expressions of key regulators involved in GSCs stemness maintenance, including CD133, CD44, and Sox2, were significantly decreased following ivacaftor treatment (Figure 2G). The above results strongly support that ivacaftor specifically and effectively impairs GSCs self-renewal ability *in vitro*.

### Ivacaftor Synergizes With TMZ to Induce Cellular Apoptosis of GSCs

**FIGURE 3:**
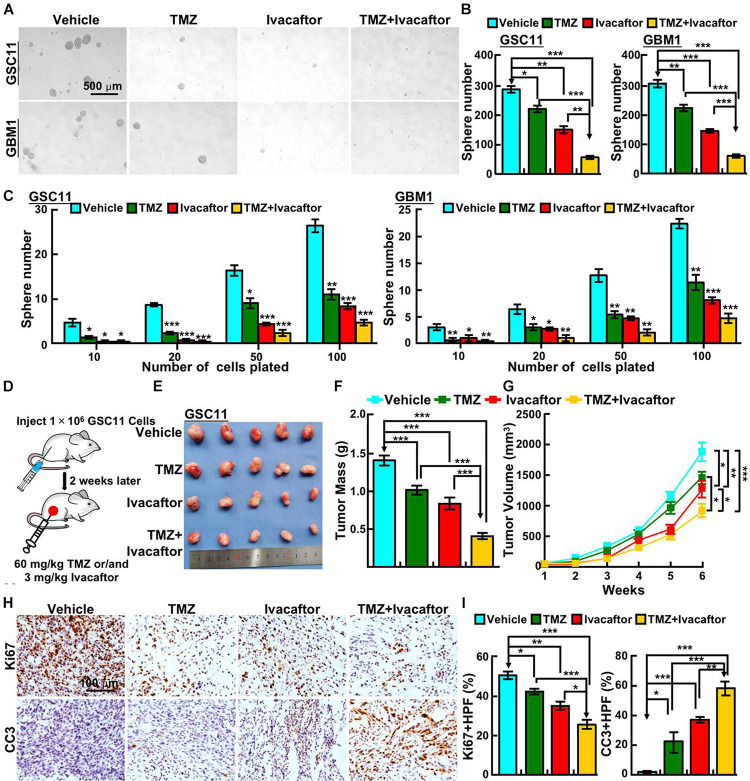
Ivacaftor sensitizes GSCs response to TMZ. **(A,B)** Ivacaftor plus TMZ decreased GSC11 (top panel) and GBM1 (bottom panel) cells tumor sphere formation ability. Scale bar: 500 μm. **(B)** Quantification result of **(A)**. **(C)** Tumor sphere derived from GSC11 (left panel) and GBM1 (right panel) cells were counted after treating indicated cells with 200 μM TMZ, 5 μM ivacaftor, or 5 μM ivacaftor combined with 200 μM TMZ. **(D)** Flow chart of xenograft tumor formation assay in nude mouse. **(E)** Representative images of tumors treated with 3 mg/kg ivacaftor alone or combined with 60 mg/kg TMZ. **(F)** Tumor masses of xenograft tumors treated with indicated drugs were shown. **(G)** Tumor volumes of xenograft tumors treated with indicated drugs were shown. **(H,I)** Representative images of immunohistochemical staining of Ki67 and CC3 in xenograft tumor sections. CC3 means cleaved caspase 3. **(I)** Quantification data for panel **(H)**. Data are shown as means ± SEM, *P < 0.05; **P < 0.01; ***P < 0.001; *t*-test.

Given that temozolomide was first-hand chemotherapy drug for GBM patients, and there are emerging references documenting that multiple compounds, including tideglusib (Zhou et al., 2016), clofoctol (Hu et al., 2019), could sensitize responses of GSCs to TMZ. We next sought to examine whether ivacaftor has similar effect. GSCs were treated with 5 μM ivacaftor, 200 μM TMZ, and 5 μM ivacaftor plus 200 μM TMZ, respectively. The tumor sphere formation ability was examined. As expected, TMZ or ivacaftor treatment significantly suppressed GSCs tumor sphere formation ability (Figures 3A,B). Importantly, ivacaftor in combination with TMZ treatment almost abrogated tumor spheres (Figures 3A,B), which was further confirmed by *in vitro* limiting dilution assay (Figure 3C).

To further validate the *in vivo* effect of ivacaftor in combination with TMZ, subcutaneous xenograft tumor model was performed. Male nude mice were randomly divided into different drug treatment groups, 1 × 10^6^ GSC11 cells were injected into nude mice subcutaneously. The mice were intraperitoneally injected with phosphate buffered saline (PBS) vehicle, ivacaftor (3 mg/kg), TMZ (60 mg/kg) and ivacaftor (3 mg/kg) plus TMZ (60 mg/kg), respectively, twice a week for 5 weeks when the xenograft tumor volume reached approximately 50 mm^3^ (Figure 3D). We found that TMZ had a modest effect on inhibiting xenograft tumor growth, whereas ivacaftor had a better inhibitory effect compared with TMZ treatment group (Figures 3E–G). As expected, TMZ combined with ivacaftor markedly suppressed xenograft tumor growth *in vivo* compared with other groups (Figures 3E–G). In line with this finding, immunohistochemistry staining showed that ivacaftor, TMZ and TMZ plus ivacaftor treatment, respectively, unanimously increased cleaved caspase 3 (CC3) positive cell staining, and decreased Ki67 positive cells staining, with the most effective phenotype in TMZ plus ivacaftor group compared to other groups (Figures 3H,I). These results prompted us to hypothesize that ivacaftor could be used as novel clinical chemotherapy strategy to cure TMZ resistant GBM patients in the future.

### Ivacaftor Suppresses Patient-Derived Xenograft Tumor Growth *in vivo*

**FIGURE 4:**
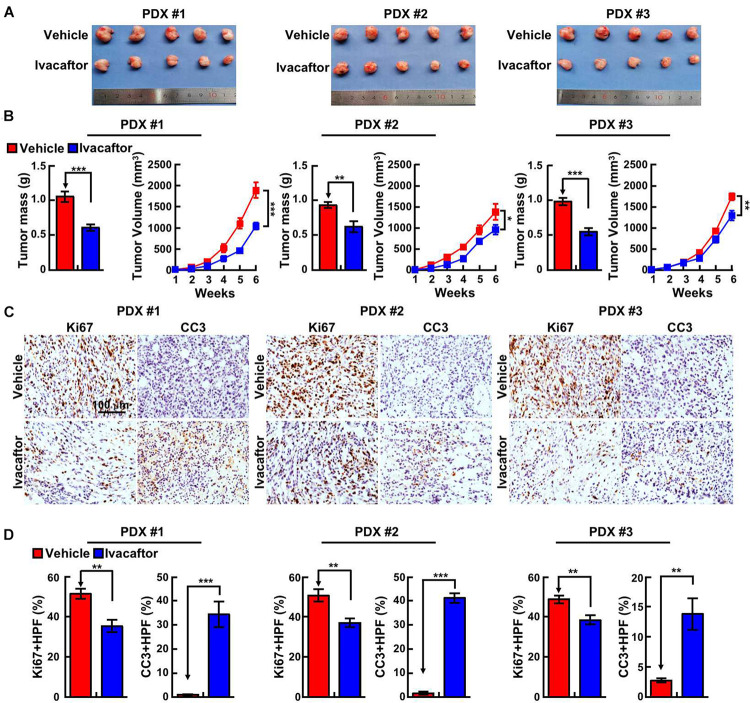
Ivacaftor inhibits PDX tumor growth *in vivo*. **(A)** Representative images of PDX tumors derived from three independent GBM clinical specimens treated with ivacaftor or vehicle. **(B)** Tumor mass and volume of three xenograft tumors treated with 3 mg/kg ivacaftor or vehicle. **(C,D)** Representative images of immunohistochemical staining of Ki67 and CC3 in three PDX tumors. **(D)** Quantification data for **(C)**. Data are shown as means ± SEM, *P < 0.05; **P < 0.01; ***P < 0.001; *t*-test.

To corroborate the clinical application of ivacaftor, patient-derived xenograft (PDX) mouse model was performed. Three clinical specimens of GBM patients were subcutaneously transplanted into nude mice and treated with ivacaftor (3 mg/kg) by intraperitoneal injection twice a week for 5 weeks. Five weeks later, the nude mice were sacrificed and the tumors were collected. As shown in Figures 4A,B, ivacaftor treatment dramatically retarded PDX tumor growth *in vivo* compared to PBS vehicle group (Figure 4B). In addition, PDX tumor sections were subject to immunohistochemical staining. The results showed that ivacaftor-treated PDX tumors exhibited more cellular apoptotic signals with increased cleaved caspase 3 staining and deceased proliferative cell markers Ki67 staining compared with the PBS vehicle treatment group (Figures 4C,D).

## Discussion

Glioblastoma is the most aggressive brain tumor in adults and characterized by high heterogeneity containing a small subpopulation of GSCs responsible for conventional therapeutic resistance and tumor recurrence (Stupp et al., 2005; Wen and Kesari, 2008). Until now, valuable and effective GSC-targeting drugs are still not available in clinical practice. It is highly notable that clinical therapies that specifically blocks crucial regulators involved in GSCs maintenance may cause better clinical outcome.

In this study, we provided the first evidence showing that ivacaftor suppresses GSCs cell proliferation, self-renewal and xenograft tumor growth, and induces cellular apoptosis. Previous study reported that FDA-approved drug ivacaftor targets cystic fibrosis transmembrane conductance regulator (CFTR) to treat cystic fibrosis, and CFTR has been indicated to facilitate glioma progression (Zhao et al., 2020). However, another finding demonstrates that CFTR activation inhibits GBM cell proliferation and migration (Zhong et al., 2019), indicating that CFTR functions as a tumor suppressor in GBM. The controversial findings of CFTR in glioma suggest that the direct target of ivacaftor in GSCs maintenance might be different. In addition, Hisert et al. (2020a,b)) provided evidence showing that ivacaftor dampens monocyte sensitivity to interferon-γ in cystic fibrosis patients. In line with this finding, ivacaftor has also been shown to improve the aberrant pathophysiology and microenvironment of the cystic fibrosis gut to favor a more healthy microbiota, which leads to reduced intestinal inflammation process (Ooi et al., 2018). Based on the fact that interferon-γ plays pivotal roles in tumor immunity (Ni and Lu, 2018), interferon-γ/JAK/STAT signaling might also mediate the inhibitory role of ivacaftor in GBM. Therefore, defining the novel target(s) of ivacaftor in GSCs using proteomic or transcriptome analysis might help us to decipher the underlying mechanism of suppressive roles of ivacaftor in GBM in the future.

A growing body of evidence indicates that CD133, CD44, and Sox2 were involved in sustaining GSCs self-renewal, which facilitates GBM tumorigenesis (Bao et al., 2006; Yin et al., 2007; Wei et al., 2013; Zhu et al., 2020). Thus, identifying leading compounds inhibit their expressions will be promising to be translated into clinical therapies for GBM patients. In this study, we showed that ivacaftor represses GSCs by directly attenuating CD133, CD44, and Sox2 expression., although the hub-gene controlling the stemness marker gene expression are not yet uncovered. However, the functional effects of ivacaftor *in vivo* using conventional xenograft tumor and PDX mouse models strongly support that the drug repurposing of ivacaftor could benefit TMZ resistant GBM patients in the future.

## Materials and Methods

### Cell Culture and Reagents

GBM1 cells were a kind gift from Dr. Yu Shi at Institute of Pathology and Southwest Cancer Center, Southwest Hospital and were characterized previously (Zhou et al., 2018). GBM1 cells were cultured in Neurobasal A medium (Gibco, 2585380) containing B27 supplement (1:50, Invitrogen, Cat#2175161), GlutaMAX (1:100, Gibco, Cat#35-50-061), Sodium pyruvate (1:100, Gibco Cat#R25-0000-Cl), MEM NEAA (1:100, Gibco, Cat#11140-050), 20 ng/mL bFGF (Gibco, Cat#PHG0266) and 1% penicillin/streptomycin. The GSC11 cells were cultured in DMEM/F12, supplemented with B27, 20 ng/mL EGF (Gibco, Cat#PHG0311L), 20 ng/mL bFGF, 4 μg/mL heparin (Sigma, H3149-500KU-9) and 1% penicillin/streptomycin. Normal human astrocyte cells (NHA) were a kind gift from Dr. Xiaozhong Peng at Institute of Medical Biology, Chinese Academy of Medical Sciences (Hu et al., 2019). NHA cells were cultured in commercial astrocyte medium (Cat#1801, ScienCell) supplemented with 1% astrocyte growth factor (ScienCell) and 2% FBS (ScienCell). All cells were cultured at 37°C in a 5% CO_2_ humidified environment. Ivacaftor and temozolomide were purchased from MedChemExpress (Ivacaftor, Cat#HY-13017), (Temozolomide, Cat#HY-17364/CS-0943).

### Cell Proliferation and BrdU Incorporation Assay

A total of 2 × 10^4^ single GSC11 and GBM1 cells were plated into laminin-precoated 12-wells plates. Exact cell numbers for each day were assessed by an automatic cell analyzer countstar (Shanghai Ruiyu Biotech Co., China, IC 1000). BrdU incorporation assay was performed as our previous indicated (Yin et al., 2017). Briefly, GSC11 and GBM1 cells were pre-treated with 10 μM BrdU (Abcam, Cat# ab142567, dilution 1:100) for 20 min, followed by fixing with 4% PFA and incubated with BrdU primary antibody (Cell Signaling Technology, Cat# 5292s, dilution 1:1000) at 4°C overnight, the cells were then gently washed with PBS and incubated with anti-mouse Alexa 594 antibody (Abclonal, Cat# 61303, dilution 1:500). DAPI was used for DNA staining. Images were examined by Nikon Ti fluorescence microscope.

### *In vitro* Limiting Dilution and Tumor Sphere Formation Assay

For *in vitro* limiting dilution assay, GSC11 and GBM1 cells were dissociated into a single-cell suspension and plated in 96-well plates (Corning, Cat#3474) in 100 μL serum-free medium. After 14 days, the numbers of tumor spheres derived from GSC11 and GBM1 cells were recorded. For tumor sphere formation assay, GSC11 and GBM1 cells were dissociated into single cells and a total of 30,000 per 2 mL serum-free medium, and then plated into ultra-low attachment 6-well plates (Corning, Cat#3471). The numbers of spheres were counted under Nikon Ti microscope.

### Flow Cytometry

Apoptotic cells in early and late stages were detected using an annexin V-APC Apoptosis Detection Kit from BioLegend (Cat#640932, PharMingen) (Xu et al., 2020). In brief, GSC11 cells were treated with indicated concentrations of ivacaftor or DMSO control. Cells were collected and washed, then resuspended by annexin V binding buffer followed by adding 5 μL annexin V-APC and 5 μL propidium iodide. Indicated cells were incubated with the staining buffer for 30 min at room temperature and analyzed by flow cytometry (BD Bioscience). More than 30,000 events were recorded for each experiment and analyzed by FlowJo software. For CD133 membrane accumulated form analysis, GSC11 cells were incubated with phycoerythrin conjugated anti-CD133 or corresponding negative control antibodies for 30 min in dark at the room temperature at 4°C, and CD133^+^ cells were then examined using flow cytometry (BD Bioscience).

### Immunoblot

Immunoblot assay was performed as documented (Jiang et al., 2018). The detail information of antibodies used in this study are as follows: β-actin (Proteintech, Cat#60008-1-1g), Bcl-2 (Cell signaling technology, Cat#15071S), PARP (Cell signaling technology, Cat#9542S), Cleaved Caspase 3 (Cell signaling technology, Cat#9661S), Bax (Abcam, Cat#ab77566), and CD133 (Miltenyi, Cat#130092395).

### *In vivo* Xenograft Tumor Model

All animal studies were approved by the Ethics Committee of Kunming Institute of Zoology. 5∼6 weeks old male BALB/c nude (GemPharmatech Co., Ltd., Nanjing, China) mice were used. For subcutaneous xenograft models, GSC11 cells (1 × 10^6^ cells per mouse) were subcutaneously injected into the nude mice. Ivacaftor (3 mg/kg) in PBS was intraperitoneally injected twice a week for 5 weeks after tumors grew to 50 mm^3^. For PDX model, three independent GBM specimens were transplanted into nude mice. Eight weeks later, the mice were sacrificed and the xenografts were cut out and transplanted subcutaneously into nude mice, mice were treated by PBS alone, TMZ alone (60 mg/kg), Ivacaftor (3 mg/kg) or TMZ plus Ivacaftor (3 mg/kg) twice a week for 5 weeks. Xenograft tumor volume were measured using Vernier calipers (Suzhou, China) by indicated time. Tumor volume was calculated by the formula length X (width)^2^/2.

### Immunohistochemistry Assay

Immunohistochemistry assay was performed as previously described (Shi Y. et al., 2019). Briefly, Immunohistochemistry was carried out in 3 μm paraffin sections (Dako, Cat#DK-2600, Glostrup, Denmark). The slides were then incubated in graded ethanol, incubated in 3% hydrogen peroxide for 20 min, and were pretreated with sodium citrate buffer for 25 min at 121°C for antigen retrieval. After washing with PBS, the tissues were immune-stained with primary antibodies against Ki67 and cleaved caspase 3 at 4°C overnight. After being washed with PBS buffer, the tissues were covered by an anti-mouse/rabbit polymer horseradish peroxidase-label for 60 min at the room temperature. The slides were treated with the prepared diaminobenzidine solution and incubated for approximate 1 min to achieve proper brown color.

### Statistics

All data are presented as the mean ± SEM. All experiments were performed at least three times. All analyses were performed using GraphPad Prism 5 (GraphPad Software). Two-tailed Student’s *t*-test was used for statistical analysis for experiments with two comparisons. *P*-values less than 0.05 was considered statistically significant. For all figures, ^∗^, ^∗∗^, ^∗∗∗^ indicate *P* < 0.05, *P* < 0.01, *P* < 0.001, respectively.

## Data Availability Statement

The raw data supporting the conclusions of this article will be made available by the authors, without undue reservation.

## Ethics Statement

The animal study was reviewed and approved by the Ethics Committee of Kunming Institute of Zoology.

## Author Contributions

CY supervised the whole study. CY and YC wrote the manuscript. KL, JP, and ZN performed the tumor sphere and xenograft tumor model assays. YS, LJ, and QW helped the real-time PCR and immunoblot assays. All authors contributed to the article and approved the submitted version.

## Conflict of Interest

The authors declare that the research was conducted in the absence of any commercial or financial relationships that could be construed as a potential conflict of interest.
